# Reconstructing individual responses to direct questions: a new method for reconstructing malingered responses

**DOI:** 10.3389/fpsyg.2023.1093854

**Published:** 2023-06-15

**Authors:** Graziella Orrù, Erica Ordali, Merylin Monaro, Cristina Scarpazza, Ciro Conversano, Pietro Pietrini, Angelo Gemignani, Giuseppe Sartori

**Affiliations:** ^1^Department of Surgical, Medical, Molecular & Critical Area Pathology, University of Pisa, Pisa, Italy; ^2^Scuola IMT Alti Studi Lucca, Lucca, Italy; ^3^Department of General Psychology, University of Padua, Padua, Italy

**Keywords:** false consensus effect, dark triad, deception, malingering, fake good, fake bad

## Abstract

**Introduction:**

The false consensus effect consists of an overestimation of how common a subject opinion is among other people. This research demonstrates that individual endorsement of questions may be predicted by estimating peers’ responses to the same question. Moreover, we aim to demonstrate how this prediction can be used to reconstruct the individual’s response to a single item as well as the overall response to all of the items, making the technique suitable and effective for malingering detection.

**Method:**

We have validated the procedure of reconstructing individual responses from peers’ estimation in two separate studies, one addressing anxiety-related questions and the other to the Dark Triad. The questionnaires, adapted to our scopes, were submitted to the groups of participants for a total of 187 subjects across both studies. Machine learning models were used to estimate the results.

**Results:**

According to the results, individual responses to a single question requiring a “yes” or “no” response are predicted with 70–80% accuracy. The overall participant-predicted score on all questions (total test score) is predicted with a correlation of 0.7–0.77 with actual results.

**Discussion:**

The application of the false consensus effect format is a promising procedure for reconstructing truthful responses in forensic settings when the respondent is highly likely to alter his true (genuine) response and true responses to the tests are missing.

## Introduction

Truthful responses to sensitive questions are difficult to collect as, under some conditions, the respondent is deceptive and responds based on social desirability. For example, pedophiles do not respond “*yes*” to direct questions such as *“are you a pedophile?.”* Similarly, drunk drivers will not admit guilt when confronted with direct questions of the “*did you do it?”* type (Locander et al., 1976). When answering such questions, most respondents express socially desirable responses or *a tendency to give overly positive self-descriptions* (Paulhus, 2002, p. 50). As a result, researchers who collect respondents’ answers at face value tend to underestimate the prevalence of undesirable characteristics while overestimating the prevalence of desirable characteristics.

The deceptive attitudes of the respondents led researchers to devise questioning techniques that guarantee complete anonymity in order to facilitate truthful responses to direct questions addressing sensitive issues. Among the best techniques proposed are the stochastic lie detector and the crosswise technique (Hoffmann and Musch, 2016). The aforementioned techniques enable respondents to conceal their responses to sensitive questions, allowing the researcher to estimate the prevalence of a sensitive characteristic across the entire sample. Although these techniques effectively determine the overall truthfulness of responses at group level, they do not address the issue of accurately estimating the truthfulness of responses for individuals. This problem occurs when a person is undergoing a psychiatric evaluation for disability or insurance purposes and is asked direct questions, such as “*Did you think about suicide*?,” as their responses may be influenced by external incentives and are collected using clinical questionnaires. Clinical questionnaires are typically built as a list of symptom-related items, and subjects respond by simulating or exaggerating their psychiatric symptoms when responding (Resnick, 1997). This behavior, called faking bad or malingering, is commonly observed in forensic settings, such as insurance claims or insanity claims in criminal proceedings (Sartori et al., 2017, 2020). Several malingering detection techniques based on validity scales (i.e., MMPI-2 and MCMI-III) or specific questionnaires (i.e., SIMS) have been proposed to detect faking in psychological testing (Young, 2014). For example, the SIMS distinguishes malingerers from honest respondents with high accuracy (Van Impelen et al., 2014), collecting responses to questions covering a wide range of pseudo-psychopathology.

However, these methods can only detect the presence or absence of the distorted response style and cannot determine whether a claimant is feigning depression or another condition. No procedure appears to be available to estimate the true level of depression in an individual after malingering has been extracted through some form of correction procedure. Rather, no model exists to retrace the truthful responses following dishonest malingering responses.

In addition to the tendency to alter a truthful response into a more severe description (malingering), the opposite phenomenon is also observed. Faking good, also known as dissimulation, is the tendency of subjects to give socially desirable responses rather than choosing responses that reflect their true feelings (Zerbe and Paulhus, 1987). In legal setting, such a tendency, independent of psychopathology, may give rise to the dissimulation of psychopathology or, in other words, denial of psychopathological symptoms (e.g., suicidal ideation). Regarding faking good on psychological questionnaires, desirability scales have been developed to assess a participant’s propensity to offer others a more desirable psychological profile (Kowalski et al., 2018). Currently, there is no procedure for reconstructing a participant’s true level of response when they provide abnormally high, socially acceptable responses on psychological questionnaires.

In this proof-of-concept paper, we will use the phenomenon known as the “*false consensus effect*” to reconstruct truthful responses on a psychological questionnaire. The false consensus effect refers to the phenomenon usually observed as overestimating the proportion of others’ responses in a given population that share characteristics with one’s own response (see Ross et al., 1977; Mullen et al., 1985, for a meta-analysis). In more detail, the prevalence estimates of OTHER questions (also called consensus estimates) may foretell overt personal behaviors. The typical format of the OTHER questions is*: “How many persons out of 100 would you guess will respond ‘yes’ to the following question? I always think about suicide.”* For example, Botvin et al. (1992) showed that peers’ smoking estimation might predict future adolescent smoking habits. The fact that people tend to base their estimates on others’ characteristics is well established despite the underlying mechanisms not being fully understood (Marks and Miller, 1987). Notably, this tendency is so strong that it persists even when people are explicitly instructed about the bias. For example, Krueger and Clement (1997) coached participants about the false consensus phenomenon just before they made their prevalence estimates and still found no reduction in false consensus (Oostrom et al., 2017). Thus, people seem unable to avoid revealing information about themselves, even when aware of exhibiting this phenomenon. It is worth noting that an alternative explanation of the false consensus effect was put forward by Dawes (1989) who explained the phenomenon using Bayesian analysis.

In the current investigation, to reconstruct truthful responses, we applied the “*false consensus effect*” as follows:

- Each participant was required to respond to the original version of the questionnaire, which required a YES/NO response (called ME questions).
- The participant was also required to respond to a variant of the original question in the following format: “*How many out of 100 persons will respond YES to the following question: Do you think about suicide?”* (OTHER% responses). The expected response is, therefore, a percentage (e.g., 10%).

The study aimed to evaluate whether it is possible to predict ME responses from the OTHER% responses at single-subject level. This prediction is expected based on the false consensus effect phenomenon. To derive accurate predictions, we applied state-of-the-art machine learning (ML) techniques (Mazza et al., 2019; Pace et al., 2019; Orrù et al., 2020a,b, 2021) as ML appears to boost predictive accuracy over more traditional psychometric techniques. Based on the consensus prevalence from the same subject to the same question, ML models were used to predict the subjects’ TRUE/FALSE responses to direct questions. As predictors, the average scores across all subjects estimating the prevalence of the TRUE response on the same item were also included.

To anticipate the results, we found that individual consensus estimates can be used to predict the participants’ own TRUE/FALSE responses to a single question with 75–80% accuracy. The same consensus prevalence estimates may be used to predict the overall percent score of the subject who answered TRUE to the same questions, with a correlation of around 0.7. Using distinct questionnaires for the evaluation of anxiety and the Dark Triad, the procedure was validated in two separate investigations.

## Study 1: anxiety

### Methods

#### Participants

For this experiment, one hundred healthy participants (79 females) were recruited using a mailing list platform. All subjects were volunteers and provided informed consent before starting the online questionnaire. The experimental procedure was approved by the local ethics committee of the University of Padua, in accordance with the Declaration of Helsinki.

The participants’ mean age was 28.9 years (27.6 females, 34.2 males), with an average of 16.4 years of education (*SD* = 2.7).

#### Stimuli and experimental procedure

The questionnaire consisted of two sections, in which participants were asked to respond twice to the same item. In the first step, participants were required to provide an estimation of the prevalence of “True” responses among their peers (OTHER%) before indicating their personal response of “True/False” to direct questions (ME responses). The subjects answered all the items in the OTHER% category first, followed by all the items in the ME category. This order was chosen because studies have shown that providing the prevalence estimation first causes a more pronounced false consensus effect (Mullen et al., 1985).

The questionnaire comprised 27 items classified as follows:

- Prevalence of Anxiety (A+): (*n* = 10) adapted from Spielberger et al. (1983). Anxious responders are expected to answer TRUE.
- Anxiety (A–): (*n* = 10) positively reframed version of the previous 10 items. Anxious responders are expected to respond FALSE to such items.
- Bizarre items (B): (*n* = 3) SIMS-adapted items (Smith and Burger, 1997). Honest subjects respond FALSE to items such as *“I forget how to get back home*,” whereas positive answers indicate that the subject is a malingerer.
- Control items (C): (*n* = 4) items that are endorsed by most subjects, such as *“I like pizza.”*

Bizarre (B) and control items (C) were required to double-check the data’s quality (Monaro et al., 2018a,b). Honest subjects were expected to respond positively to control items (C) and negatively to bizarre items (B).

When responders take a test in a real setting in which there is fake proneness (e.g., insurance claims) it has been suggested (Van Impelen et al., 2014) that items that are rarely endorsed by responders or items that are frequently endorsed by responders should be included in order to evaluate the overall level of accuracy of the responders. For this reason, we also included Bizarre (B) and Control items (C).

#### Data analysis

ML techniques implemented in the Weka software (Frank et al., 2016) were used to analyze the data. Weka is a Java-based scripting language-based collection of ML algorithms for data mining tasks. It includes different tools for data preparation, classification, regression, clustering, association rule mining, and visualization.

We used 10-fold cross-validation to test ML models in order to obtain realistic estimates of single-subject single question responses to ME questions. Cross-validation is usually a very good procedure for determining the extent to which a result is replicable, at least for what has been referred to as exact replication (Cumming, 2008). When no cross-validation is used, the results are inflated and overly optimistic, and the model may not replicate when applied to out-of-sample data (Bokhari and Hubert, 2018). We applied: (1) ML classification techniques to predict the specific ME response (TRUE/FALSE) of a participant based on their prevalence estimation on OTHER%; (2) ML regressors to predict the percentage of TRUE responses of a single participant given to a set of items (A+; A-, C; B).

### Results of Study 1

The analysis was carried out on the entire set of 27 questions (one for OTHER% and one for ME). The results were obtained by analyzing 2,700 stimuli for the OTHER% questions and 2,700 for the ME questions. The percentage of TRUE and FALSE ME responses by item type is presented in Table 1. As expected, a few participants endorsed bizarre (B) items, socially undesirable items, while the majority endorsed control items (C). True responses to A+ and A-items were around 50, 70% for control items (C), and 13% for bizarre items (B).

**Table 1:** True and False responses to items indexing A+, A–, C, and B.

Total responses	TRUE (*n* = 1,274)	FALSE (*n* = 1,434)	Total responses
A+	461 (46%)	541 (53.9%)	1,002
A–	492 (49.1%)	510 (50.8%)	1,002
C	282 (70.14%)	120 (29.8%)	402
B	39 (12.9%)	263 (87%)	302

Control questions and bizarre questions were included to scan the full range of endorsement by participants.

In Table 2, average OTHER% estimations are reported separately for TRUE responses to ME questions and FALSE responses to ME questions. The magnitude of the effect size (*d* = 1.2) indicated that the OTHER% estimations differed based on whether the subject endorsed the item when responding to ME questions. For the effect size interpretation, Cohen (1988, 1995) specifies the following intervals: 0.1–0.3 for a modest effect, 0.3–0.5 for an intermediate effect, and 0.5 and above for a large effect.

**Table 2:** Columns report the average (SD) of OTHER% and MEANOTHER%, given TRUE and FALSE ME responses to direct questions.

Average % of OTHER% estimation given	TRUE avg. (*SD*)	FALSE avg. (*SD*)	Effect size (*d*) ME vs. OTHER	Correlation (r) with TRUE/FALSE ME responses
OTHER%	0.620 (0.22)	0.363 (0.21)	*d* = 1.2	*r* = 0.506
MEANOTHER%	0.559 (0.12)	0.394 (0.16)	*d* = 1.5	*r* = 0.512

The OTHER% estimations of TRUE depend on which ME response was given by the participant.

The correlation between the TRUE/FALSE ME response and OTHER% was *r* = 0.506. MEANOTHER% indicated the average value of the same figure across all participants, with a correlation of 0.512 to individual ME responses. The value of Pearson r correlation varies between –1 (a perfect negative correlation) to +1 (a perfect positive correlation) (Pearson, 2008).

From the data reported in Table 2, a strong false consensus effect emerged. Participants who gave a high percentage in OTHER% estimates also endorsed the corresponding sentence when responding to the ME questions. Those who answered TRUE had a significantly higher percentage of estimation in OTHER% (62%) than those who answered FALSE (36%).

#### Prediction of the specific ME (TRUE/FALSE) response based on OTHER% and MEANOTHER% prevalence estimation

**Figure 1:**
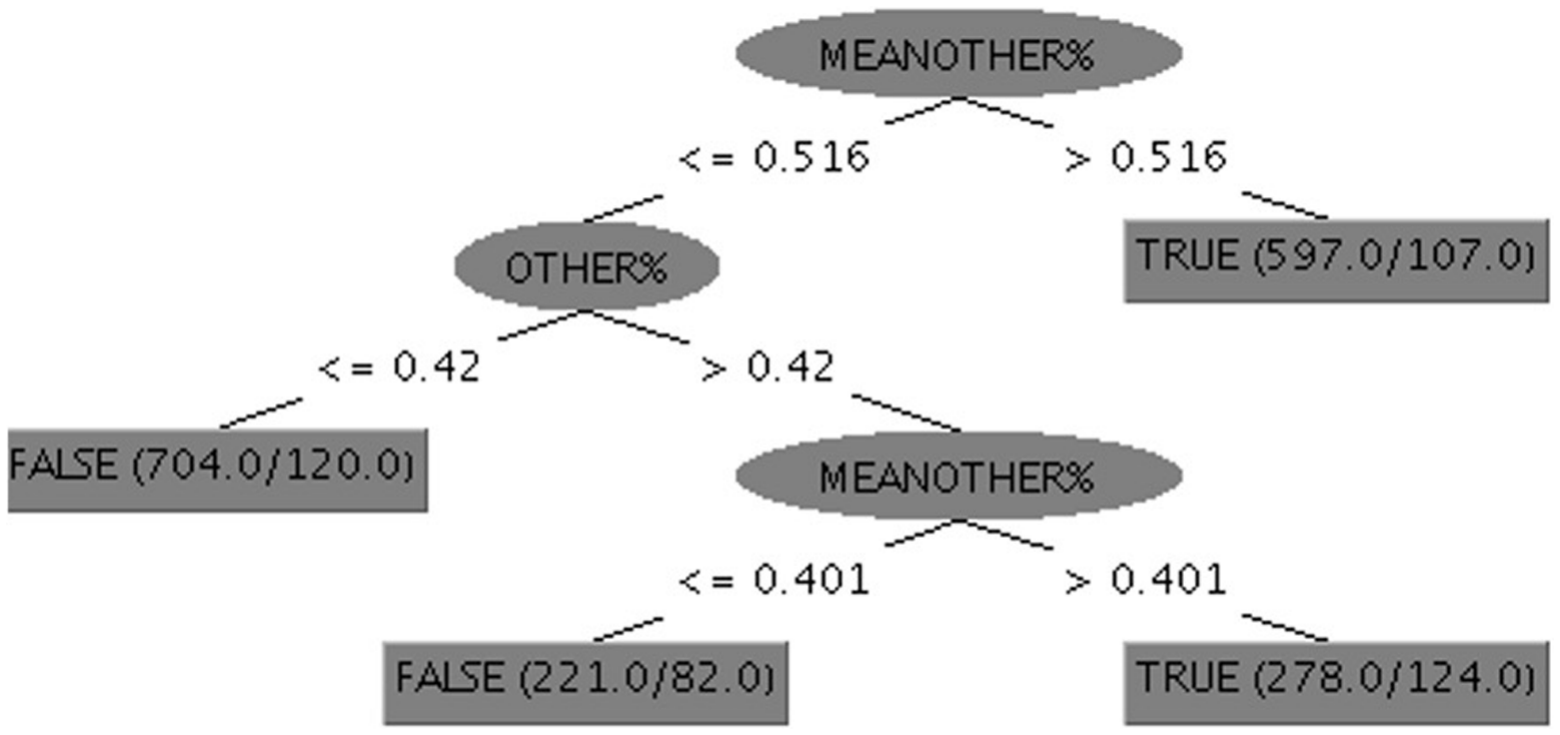
C4-5 decision tree. The final leaf reports the number of instances classified by the rule and the number of errors. For example, the first node is the following: IF MEANOTHER%>0.516, THEN the response predicted by this leaf is TRUE with an accuracy = 82% (597 responses are in this leaf with 107 errors).

We showed that the percentage of ME responses can be predicted using OTHER% responses. A Naive Bayes classifier^1^ was trained and validated to classify a single response to a single item as TRUE or FALSE on the basis of the estimation of OTHERS% and the MEANOTHER%. The first value was the participant’s estimation of the percentage of peers expected to endorse the item (e.g., How many of 100 people would respond TRUE to the following question: “*I forget how to get back home”* → 12%). MEANOTHER% indicated the average value of the same figure for all participants on the target question. By comparing the responses of the participants and all other responses, this information contributed to the classification. Results of the Naive Bayes classifier trained using the 10-fold cross-validation were the following: correctly classified items = 2061/2700, accuracy = 76.33%, with a mean absolute error (MAE) = 2.97% and AUC = 0.84. In Table 3, the confusion matrix derived from cross-validation is reported. The good result generalizes also to other classifiers based on different statistical assumptions (e.g., Logistics, SVM, Decision Tree). The results of these classifiers are comparable to the figures reported in detail above. This result indicates that the result is robust across models and is not the result of model hacking (cherry-picking the best-performing model, Orrù et al., 2020a). Best-performing models are usually difficult to interpret, giving rise to a clear interpretability/accuracy trade-off (Johansson et al., 2011). In short, interpretable models usually are not the best performers, and the best performer’s classifiers are usually not interpretable. One strategy consists of using hard-to-interpret ML models to estimate maximum accuracy and easy-to-interpret decision rule models for more confidence-based evaluations. One such classifier is the C4-5 decision tree (Quinlan, 1993) which output is a set of easy to understand if-then decision rules. Running the C4-5 decision tree algorithm, we identified the decision tree represented in Figure 1. The three decision rules reported above yielded an accuracy of 75.78% and an AUC = 0.803.

**Table 3:** Confusion matrix derived from cross-validation.

Classified/Actual (*n* = 2,700)	TRUE	FALSE
TRUE	882 (69.4%)	251 (17.5%)
FALSE	388 (30.5%)	1,179 (82.44%)
Total	1,270	1,430

The AUC’s value ranges from 0 to 1. A model with 100% incorrect predictions has an AUC of 0.0, while a model with 100% correct predictions has an AUC of 1.0. In general terms, an AUC of 0.5 often indicates no discrimination in a forced choice between two alternatives discrimination. A value of AUC from 0.7 to 0.9 is considered good, more than 0.9 is regarded as remarkable (Carter et al., 2016).

#### Prediction of the percentage of TRUE responses of a single participant for a given set of items (i.e., A+; A–; C; B)

The above-mentioned analysis reported above was carried out for all of the stimuli presented to the participants. The ML models were run again separately on A+, A-, B, and C items. Similar results were observed, separately, for anxiety-related items, as well as bizarre (B) and control (C) items.

We evaluated the prediction of the percentage of TRUE responses (%TRUE) to ME questions given by each participant to a set of items belonging to A+, A–, C, and B. For every participant, four TRUE% scores have been computed, one for each item category (A+, A–, C, and B), and the regression model was developed to predict this value based on item type (A+, A–, C, and B), OTHER%, and MEANOTHER%. A linear ridge regression model (using 10-fold cross-validation) yielded a correlation between the actual and predicted score of *r* = 0.719 with a mean absolute error (MAE) of 0.168. A similar result was observed using only average OTHER% and MEANOTHER% (without any information about the item type) as a correlation of *r* = 0.72 and MAE of 0.17, resulting from a similar ridge regression model. This result indicates that the same model can accurately predict the ME responses independently from their content (A+, A–, C, and B). Finally, the results obtained by different regressors (MLP, SVM regressor) indicate that the reported result was robust across regressors based on differing assumptions.

#### Prediction of the percentage of TRUE responses of a single participant for a given set of items (i.e., A+; A–)

In order to evaluate that the prediction accuracy is not inflated by prediction on control items B and C, we have replicated the analysis using only the A+ and A-items. The following linear regression analyses was conducted with 10-fold cross-validation, considering the items Anxiety (A+) and Anti-anxiety (A–). The results indicate that the correlation between the true and estimated data (ME TRUE %) was *r*^2^ = 0.7246, *p* < 0.001 (mean absolute error = 0.1674). The same result was confirmed with other regressor models (Support Vector Machine).

In addition, the possibility of inferring a subject’s single response to individual items (ME TRUE or ME FALSE) from the OTHERS estimate made by the group was investigated. Individual ME responses were predicted (using a Random Forest classifier) with 77.7% accuracy on the basis of OTHER% estimates.

## Study 2: Dark Triad

The Dark Triad is a group of three sub-clinical personality traits generally seen as negative and socially undesirable. The three traits are: (1) Narcissism, which is characterized by excessive self-importance, a lack of empathy for others, and a need for admiration; (2) Machiavellianism, which involves a tendency to be manipulative and exploit others for personal gain; (3) Psychopathy, which is characterized by a lack of remorse or guilt, a tendency to be deceitful and manipulative, and a lack of empathy and concern for others. These traits are often referred to as the “Dark Triad” because they are associated with negative and socially undesirable behaviors (Paulhus and Williams, 2002). A super-short version of the Dark Triad psychometric questionnaire has been proposed (Dirty Dozen; Jonason and Webster, 2010) and was used here.

### Methods

#### Participants

Overall, 87 participants took part in this online study (37 females and 50 males). Their average age was 48.4 years (SD = 9.03; range: 28–71). The educational level was of 18 years (SD = 1.5, Range: 15–22). As in the previous experiment, participants volunteered to complete the online questionnaire under the experimenter’s supervision and provided informed consent.

#### Stimuli and experimental procedure

The questionnaire included 31 questions. It was constructed using an Italian version of the Dirty Dozen (Jonason and Webster, 2010). In addition to the canonical 12 items of the original questionnaire (DD), we added 12 items from the brand-new construct of the Light Triad (LT) (Kaufman et al., 2019), which may be considered the opposite of the Dark Triad. Seven control items (C) were added, with the expectation that they would elicit a high or low percentage of true responses (e.g., *“I like pizza”* and *“I do not know the days of the week,”* respectively). Control items are items that are endorsed or rejected by a high number of responders but are unrelated to the psychological dimension investigated by the Dark and Light triad. Each item was presented twice, as in Study 1. The first presentation required an estimation of the percentage of people expected (OTHER%) to endorse the item by the respondent. The second required the participant to indicate their personal response of “True/False” to direct questions (ME responses). The participant was required to click on one of the 10% range (from 0 to 100%) boxes appearing beneath the presented item. This method of collecting the participant’s prevalence estimation differs from the procedure used in Study 1, which was a sliding cursor. After all the 31 questions were presented in the above format, the participant was required to give his/her (ME) response (e.g., *I am an honest person:* TRUE/FALSE) to all questions.

#### Data analysis

The data analysis procedure was the same as in Study 1. All results reported are based on a 10-fold cross-validation procedure.

### Results of Study 2

The total number of responses collected and analyzed for OTHER% prevalence estimation was 2,697 (31 total questions, presented to 87 participants) and 2,697 ME responses. For all items, the TRUE responses to direct questions were 55%. When participants responded TRUE, their estimation of the percentage of TRUE responses on OTHERS% was, on average, 60.89%, whereas when responding FALSE, the responses TRUE were 29.8% (*d* = 1.203). The correlation between OTHER% and TRUE/FALSE was 0.52, and between MEANOTHER% and TRUE/FALSE was 0.61. As observed in Study 1, a strong consensus effect emerged: participants endorsed more ME items when the corresponding OTHER prevalence estimates were higher, suggesting that they were influenced by their own responses while estimating others.

#### Prediction of the individual ME (TRUE/FALSE) response based on OTHER% and MEANOTHER% prevalence estimation

The strong false consensus effect permitted to predict individual responses on the basis of OTHERS% estimations. Classification accuracy obtained using the Naive Bayes classifier was 77.60% (*n* = 2093/2697) with an AUC = 0.87. The confusion matrix of the out-of-sample classifications, obtained using the Naïve Bayes classifier reported above, is shown in Table 4. Similar classification accuracies were obtained with other classifiers, indicating that the prediction was not dependent on specific assumptions made by a specific classifier (Salzberg, 1994). The analysis was conducted for all 31 stimuli presented to the participants. Still, separate analyses are reported for control items (C), for Dirty Dozen items (DD), and for Light Triad items (LT) to determine whether the same results could be observed for items belonging to the same category.

**Table 4:** Confusion matrix derived from cross validation.

Classified/Actual (*n* = 2,697)	TRUE	FALSE
TRUE	1,153 (80.2%)	321 (25.4%)
FALSE	283 (19.7%)	940 (74.5%)
Total *n* = 2,697	1,436	1,261

The data are collected only from out-of-sample testing in 10-fold cross-validation.

#### Prediction of the percentage of TRUE responses of a single participant for a given set of items (i.e., control, DD, LT)

The total data collected for each participant was 261 (87 participants corresponding to the TRUE% responses to DD, LT, and control, for 261 examples). To predict the percentage of TRUE responses to the ME questions for each participant, the first set of regressors was evaluated using as predictors the type of question (C, DD, LT), together with OTHER% and MEANOTHER%. The results for the regressors are reported in Table 5. Irrespective of the regressor, the correlation between the actual and predicted value was around 0.77. In this specific case, removing the question type (C, DD, LT) from the predictors resulted in a slightly lower correlation. Corresponding importance of using question type as input was not observed in Study 1.

**Table 5:** Correlation between the actual and the predicted value of the TRUE% response for different regressors.

Regressor	Correlation Predictors/Question type/ OTHER%/MEANOTHER%	Correlation Predictors/OTHER%, MEANOTHER%
Ridge regressor	0.770	0.624
MLP regressor	0.808	0.700
SVM regressor	0.776	0.624
M5P	0.774	0.648

The two columns report models developed with and without the question type (Control, DD, LT) as predictor. MP5 was included as representative of a regressor based on decision trees (Wang and Witten, 1997).

## Discussion

We investigated the feasibility of using ML-based methods to reconstruct answers to direct questions on sensitive topics (such as anxiety evaluation and undesirable personality traits) based on the consensus estimations obtained from indirect questions on the same subject.

Two experiments were designed as follows: first, participants had to estimate the prevalence of TRUE responses on a given item among peers (the subjective estimation of the percentage of YES responses that a group of 100 people will endorse). The same subject was then asked to give his own response (TRUE/FALSE) to the same question. Two studies were undertaken with a total of 187 individuals, and two questionnaires were used to get at two very different clinically relevant topics: anxiety and the dark triad. These issues are likely to elicit faked responses in opposite directions. In medico-legal settings, claimants are prone to either fake bad to aggravate their symptoms (to have an advantage), or to fake good, trying to hide personality traits or behaviors that would lead them to a disadvantage, such as in child-custody claims. The items addressing anxiety and the Dark Triad were adapted from standard questionnaires that included clinical (anxiety) and subclinical (Dark Triad) personality characteristics. To include positive and negative verbal expressions of the same psychological construct, items addressing the same issues were framed reversely.

Taking all results together, we have found that individual responses to single direct questions requiring TRUE/FALSE answers can be reconstructed with 75–88% accuracy using the subject’s estimate of consensus on the same question as the group’s corresponding estimation. Accurate prediction is possible at group level and individual subject level, as individual responses to a single direct question can be reconstructed, through the ML algorithm, with an accuracy of 75–80%, depending on the issue under investigation. ME responses to some issues (e.g., bizarre items, *I forget how to get back home*) are predicted more successfully than others (e.g., anxiety items). These findings suggest that consensus estimation can be used to reconstruct subject responses regardless of the item’s content. Prevalence estimation among peers has the ability to predict single-item responses as well as total scale scores.

Machine learning regressors were developed, and the predicted value of ME responses based on OTHER% responses correlate with the observed values by 0.72–0.77. The “*false consensus effect*” describes a correlation between the participant’s actual response and his estimate of his peer responses to the same question. If the subject’s response is TRUE, he will estimate that most subjects will respond in the same way. If his response is FALSE, he thinks that most subjects will endorse his FALSE response. This phenomenon emerges clearly in our data, with an effect size around *d* = 1 (large effect) on almost all items.

The results obtained using this technique may aid in resolving the problem of reconstructing true responses when responses to direct questions are deceptive and distorted by internal incentives such as social desirability or external incentives, as well as in compensation claims. The possibility of indirectly and accurately predicting individual responses to direct questions based on indirect estimation of peer responses to the same question opens promising avenues for studying truthful responses to sensitive issues.

In summary, prevalence estimation may be used to reconstruct truthful responses to potentially deceptive items, allowing us to determine whether the examinee faked the test and his true score. These experiments support the idea that the false consensus effect could be at the core of future psychometric questionnaires that could help us identify genuine answers from examinees who could be at risk of faking.

The present investigation suffered from a number of limitations. Even though with ML techniques, the items presented to the algorithm consisted of every single item repeated twice for each subject, providing a thousand observations for each experiment, the sample size could have been greater. Future studies should look the sample size and enlarge its variability. Even if age and education did not correlate with the type of answers given or the consensus estimates, future research should look into whether a more balanced (for instance less educated and more males) would have produced different results.

The use of the false consensus effect in forensic settings is a promising procedure for reconstructing truthful responses, and the studies reported here indicate that the participant response to single items may be estimated accurately using an indirect method.

However, the method’s efficiency in reconstructing truthful responses has yet to be evaluated in high stake faking, when the participant has a clear advantage or disadvantage in successfully hiding the true response. The studies reported here, in fact, did not require the subject to alter their response intentionally and, in some way, can be considered as preliminary research for the development of a method for reconstructing the true response to questions to which the subject answers in a presumably less transparent manner.

## Data availability statement

The raw data supporting the conclusions of this article will be made available by the authors, without undue reservation.

## Ethics statement

The studies involving human participants were reviewed and approved by the Ethics Committee of UNIPD. The patients/participants provided their written informed consent to participate in this study.

## Author contributions

GO, GS, and EO: conceptualization. EO: data curation and investigation. GS and GO: formal analysis. GS: methodology and supervision. GO, EO, MM, CS, CC, PP, AG, and GS: writing – review and editing. All authors contributed to the article and approved the submitted version.

## Conflict of interest

The authors declare that the research was conducted in the absence of any commercial or financial relationships that could be construed as a potential conflict of interest.

## Publisher’s note

All claims expressed in this article are solely those of the authors and do not necessarily represent those of their affiliated organizations, or those of the publisher, the editors and the reviewers. Any product that may be evaluated in this article, or claim that may be made by its manufacturer, is not guaranteed or endorsed by the publisher.

## Footnotes

^1^The Naive Bayes algorithm is a method that uses the probabilities of each feature (independent variable) predict the class the individual case belongs to. It is referred to as “naive” because all features are regarded as independent, which is rarely the case in real life. Naive Bayes simplifies the calculation of probabilities by assuming that the probability of each attribute belonging to a given class is independent of all other attributes. This is a strong and frequently false assumption, but it results in a fast and effective classification method. Despite the apparently unrealistic assumptions, it has been shown the mathematical properties of the good performance of the classifier.
